# Assessment of MALDI-TOF mass spectrometry for filariae detection in *Aedes aegypti* mosquitoes

**DOI:** 10.1371/journal.pntd.0006093

**Published:** 2017-12-20

**Authors:** Djamel Tahir, Lionel Almeras, Marie Varloud, Didier Raoult, Bernard Davoust, Philippe Parola

**Affiliations:** 1 Unité de Recherche en Maladies Infectieuses et Tropicales Emergentes (URMITE), Aix-Marseille Université, UM63, CNRS 7278, IRD 198 (Dakar), Inserm 1095, AP-HM Institut Hospitalo-Universitaire Méditerranée Infection, Marseille, France; 2 Unité de Parasitologie et Entomologie, Département des Maladies Infectieuses, Institut de Recherche Biomédicale des Armées, Marseille, France; 3 Ceva Santé Animale SA, Libourne, France; Universidade Federal de Minas Gerais, BRAZIL

## Abstract

Matrix Assisted Laser Desorption/Ionization Time-of-Flight Mass Spectrometry (MALDI-TOF MS) is an emerging tool for routine identification of bacteria, archaea and fungi. It has also been recently applied as an accurate approach for arthropod identification. Preliminary studies have shown that the MALDI-TOF MS was able to differentiate whether ticks and mosquitoes were infected or not with some bacteria and *Plasmodium* parasites, respectively. The aim of the present study was to test the efficiency of MALDI-TOF MS tool in distinguishing protein profiles between uninfected mosquitoes from specimens infected by filarioid helminths. *Aedes aegypti* mosquitoes were engorged on microfilaremic blood infected with *Dirofilaria immitis*, *Brugia malayi* or *Brugia pahangi*. Fifteen days post-infective blood feeding, a total of 534 mosquitoes were killed by freezing. To assess mass spectra (MS) profile changes following filariae infections, one compartment (legs, thorax, head or thorax and head) per mosquito was submitted for MALDI-TOF MS analysis; the remaining body parts were used to establish filariae infectious status by real-time qPCR. A database of reference MS, based on the mass profiles of at least two individual mosquitoes per compartment, was created. Subsequently, the remaining compartment spectra (N = 350) from *Ae*. *aegypti* infected or not infected by filariae were blind tested against the spectral database. In total, 37 discriminating peak masses ranging from 2062 to 14869 daltons were identified, of which 17, 11, 12 and 7 peak masses were for legs, thorax, thorax-head and head respectively. Two peak masses (4073 and 8847 Da) were specific to spectra from *Ae*. *aegypti* infected with filariae, regardless of nematode species or mosquito compartment. The thorax-head part provided better classification with a specificity of 94.1% and sensitivity of 86.6, 71.4 and 68.7% of *D*. *immitis*, *B*. *malayi* and *B*. *pahangi* respectively. This study presents the potential of MALDI-TOF MS as a reliable tool for differentiating non-infected and filariae-infected *Ae*. *aegypti* mosquitoes. Considering that the results might vary in other mosquito species, further studies are needed to consolidate the obtained preliminary results before applying this tool in entomological surveillance as a fast mass screening method of filariosis vectors in endemic areas.

## Author summary

Filariosis is a disease group affecting humans and animals, caused by nematode parasites of the family Onchocercidae, superfamily Filarioidea. These parasites can be transmitted, essentially, by mosquitoes during blood meals of infected female specimens. Screening vectors for these filariae currently relies on time- and resource-consuming methods such as dissection and polymerase chain reaction-based methods. Here, we applied matrix-assisted laser desorption/ionization time-of-flight mass spectrometry (MALDI-TOF-MS) to assess whether this tool can detect changes in the protein profiles of *Aedes aegypti* infected with filarioid helminths compared to those uninfected by testing different parts of mosquitoes. First a reference mass spectra database from *Ae*. *aegypti* infected or not infected by filariae was created using MS from 47 specimen compartments. Then we tested the remaining mass spectra (350 x 4) in a blind validation test. Regardless of filariae species, the best correct classification rate was obtained from the thorax-head part with a specificity of 94.1% and sensitivity of 86.6, 71.4 and 68.7% for non-infected and *D*. *immitis*, *B*. *malayi* and *B*. *pahangi* infected mosquitoes respectively. The results indicated that MALDI-TOF MS is potentially able to screen *Aedes aegypti* mosquitoes as being non-infected or filariae-infected. Furthermore, complementary works using other mosquito species infected with different filarioids are needed to reinforce these preliminary results prior to apply this tool on field samples.

## Introduction

Mosquitoes are blood-sucking arthropods with a global distribution. They represent a huge threat to humans and animals as vectors of pathogens [1,2]. In passing from host to host, some mosquito species may transmit parasitic diseases (*i*.*e*. malaria, lymphatic filariosis and dirofilariosis), arboviroses (*i*.*e*. dengue, west nile, zika, eastern equine encephalitis disease and others) [3,4], and possibly bacterial diseases (*i*.*e*. *Rickettsia felis* infection) [5].

Dirofilarioses due to *Dirofilaria immitis* and *Dirofilaria repens* are mosquito-borne parasitic infections of dogs and other wild carnivores, which function as reservoirs. Humans and cats are less suitable hosts [6,7]. *D*. *immitis* has a worldwide distribution and it is endemic in tropical and temperate regions throughout the world, whereas *D*. *repens* is exclusive to the Old World [8]. Lymphatic filariosis (commonly known as elephantiasis) is a neglected human borne-disease caused by infection with three different filarioid worms. Most of the infections worldwide are caused by *Wunchereria bancrofti* [9]. However, in Asia the disease can also be caused by *Brugia malayi* and *Brugia timorii* [10]. *Brugia pahangi*, another zoonotic lymphatic filarioid nematode that is naturally found in cats but also found in other types of hosts, can cause clinical infection in humans, with clinical presentations that are consistent with lymphatic filariosis [11]. All these filariae parasites have biphasic life cycles involving the definitive mammalian host and various genera of mosquito vectors, including *Aedes*, *Anopheles*, *Culex*, *Mansonia*, and *Ochlerotatus* [7,12].

The capture and identification of mosquitoes, as well as the detection of associated pathogens, are important steps for monitoring mosquito-borne diseases like dirofilariosis and lymphatic filariosis. Mosquito identification is performed using mainly morphological keys and/or molecular methods [13]. However, screening the mosquitoes according to their filarioid infection rate is based on dissecting freshly killed, individual female mosquitoes. In fact, mosquito dissection is considered the gold standard for measuring infection rates and densities in the vector [14]. However, this is a labor-intensive and time-consuming procedure requiring entomological expertise [15,16]. Molecular methods such as PCR and gene sequencing have been developed as a tool for detecting filarioid parasite DNA in mosquitoes. These methods have been applied as molecular xenomonitoring of filariosis [17]. However, molecular techniques are relatively expensive. But, sometimes, for economic reasons, it is not possible to routinely use molecular biology as a monitoring tool for mosquito vectors. Therefore, a faster and more cost-effective technique for the simultaneous identification of mosquito vector species and detection of their associated pathogens could improve entomological surveillance of mosquitoes and mosquito-borne diseases.

MALDI-TOF MS has been introduced as a routine method in diagnostic microbiology laboratories for identifying bacteria, archaea and fungi isolated from different samples [18,19]. More recently, this proteomic approach has been used with success in the identification of arthropods such as mosquitoes, fleas and ticks [13]. In addition, two preliminary studies showed the ability of MALDI-TOF MS to differentiate ticks infected or not infected with *Borrelia crocidurae* or *Rickettsia* spp. using specimen legs [20,21]. Finally, MALDI-TOF MS showed a good performances of specificity (100%) and sensitivity (92%) when this tool was applied to screen mosquitoes infected or not infected with *Plasmodium berghei* protozoan parasites [22]

The aim of this study was to determine MALDI-TOF MS’s effectiveness in detecting changes in the protein profiles of *Ae*. *aegypti* mosquitoes infected with filarioid helminths compared to uninfected ones.

## Materials and methods

### Ethics statement

*Aedes aegypti* (Black-eyed Liverpool strain) were artificially infected by feeding them on a membrane feeder which contained blood with microfilariae, as previously described [23]. This experiment was conducted at TRS Labs, Inc. in Athens, Georgia (USA) under AUP 15–07 (2). The protocol was approved by the laboratory's Institutional Animal Care and Use Committee (IACUC) prior to the study beginning.

### Experimental model

*D*. *immitis*, *B*. *malayi* and *B*. *pahangi* infected and non-infected *Ae*. *aegypti* were provided by TRS Labs, Inc. in Athens, Georgia (USA). For each nematode species two experimental groups of four- to six-day-old female mosquitoes were constructed: one infected group in which mosquitoes were fed with microfilaremic blood and one control non-infected group in which mosquitoes were feed with non-microfilaremic blood. All mosquitoes were starved for 24 hours prior to blood feeding. In brief, *D*. *immitis* microfilaremic blood were collected from naturally infected dog into syringes containing 3.8% sodium citrate. Mosquitoes were fed for at least 1 hour using an artificial feeding system (Hemotek feeding system; Discovery Workshops, Lancashire, United Kingdom) [24] loaded with 3 mL of infected (5,000 mf/ml) or amicrofilaremic blood containing sodium citrate anticoagulant (control). While, for *B*. *malayi* and *B*. *pahangi* infected or uninfected mosquitoes, female *Ae*. *aegypti* were allowed to feed for 40 mins on anaesthetized, infected or uninfected (control) jirds, *Meriones unguiculatus* with microfilaremiae of *B*. *malayi* or *B*. *pahangi* ranging from 192–1,008 mf/20 mL blood. After the blood meal, all mosquitoes were fed on 10% sucrose solution and kept under standard laboratory-rearing conditions for 15 days, the timeframe necessary for the mosquito parasite cycle. Subsequently, mosquitoes were killed by putting them in dry ice and stored at– 20°C for subsequent analysis.

### Mosquito analysis

#### Mosquito screening for filarioid helminths using real-time PCR

Each mosquito was successively washed in 70% ethanol and sterile water for 10 mins, before being dried on sterile filter paper. Molecular analysis was done to establish the infectious status of mosquitoes engorged on filariae infective blood (Table 1). Mosquito body parts (legs, heads, thoraces or heads and thoraces) selected for MS analysis were classified in groups one to four, respectively. For each group, the remaining body parts were used to determine their filariae infection status by qPCR.

**Table 1:** Classification of samples submitted to MALDI-TOF MS and real-time PCR results.

	Compartment	Number of specimens submitted to MS	qPCR+ (%)*
***Aedes aegypti* fed on microfilaremic-free blood (control group, n = 98)**	legs	40	/
	thorax	15	/
	thorax-head	22	/
	head	21	/
***Aedes aegypti* fed on *D*. *immitis* microfilaremic blood (n = 161)**	legs	75	58 (77.3)
	thorax	23	17 (73.9)
	thorax-head	63	18 (28.5)
	head	/	/
***Aedes aegypti* fed on *Brugia malayi* microfilaremic blood (n = 143)**	legs	60	54 (90)
	thorax	44	42 (95.4)
	thorax-head	16	10 (62.5)
	head	23	22 (95.6)
***Aedes aegypti* fed on *Brugia pahangi* microfilaremic blood (n = 132)**	legs	79	66 (83.5)
	thorax	15	12 (80)
	thorax-head	24	19 (79.1)
	head	14	12 (85.7)
**Total**		**534**	**330**

*qPCR were done on the remaining body part from MS analysis, for each specimen to establish filariae infectious status.

For each mosquito, the carcass not used for MS analysis was transferred to a 1.5 mL micro-centrifuge tube and crushed in 180 μL buffer G2 (Qiagen, Hilden, Germany) used for molecular filariae detection. Then 20 μL of proteinase K (20 mg/mL; Qiagen, Hilden, Germany) was added to the ground mosquito body and the mixture incubated overnight at 56°C to ensure complete lysis of the tissue. Whole genomic DNA was extracted in 50 μL of Tris EDTA (TE) buffer using the EZ1 DNA Tissue kit (Qiagen, Hilden, Germany) according to the manufacturer's instructions. The DNA was stored at –20°C until the sample was used for qPCR.

For each group, all DNA samples were individually screened for the presence of *D*. *immitis*, *B*. *malayi* or *B*. *pahangi* by qPCR as previously described [25,26]. In brief, the real-time PCR experiment was performed in a total reaction volume of 20 μL, containing 10 μL master mix Takyon (Eurogentec France, Angers, France), 3.5 μL distilled water, 0.5 μL (20 μM) of each primer, 0.5 μL probe (5 μM), and 5 μL DNA template. All amplifications in real-time PCR were performed on the thermal cycler CFX96 Touch detection system (Bio-Rad, Marnes-la-Coquette, France). For each reaction, DNA-free water and DNA from uninfected mosquitoes were used as negative controls. *D*. *immitis*, *B*. *malayi* and *B*. *pahangi* DNA were used as positive controls.

#### Sample preparation for MALDI-TOF MS analysis

The volumes of supplying buffers for sample homogenization were adjusted according to the body part used: 15 μL of 70% (v/v) formic acid (Sigma) plus 15 μL of 50% (v/v) acetonitrile (Fluka, Buchs, Switzerland) for mosquito legs (Nebbak et al, 2016) and 30 μL of 70% formic acid plus 30 μL of 50% acetonitrile for heads, thoraces and heads plus thoraces. A pinch of glass powder (Sigma) was added to each sample and FastPrep-24 (MP Biomedicals Santa Ana, California, USA) automated grinding methods were used for sample destruction. The FastPrep-24 parameters were 4 cycles at 5 m/s for 40 secs for legs and 6 cycles at 5 m/s for 40 secs for the three others body parts. The homogenate was then centrifuged at 6,700 x g for 30 seconds and one microliter of the supernatant of each sample was spotted in quadruplicate onto the polished-steel MSP 96 target plate (Bruker Daltonics, Bremen, Germany). The spots were dried at room temperature for a few minutes before being covered with 1 μL of matrix solution containing saturated α-Cyano-4-hydroxy-cinnamic acid (CHCA) (Sigma), 50% acetonitrile (Sigma), 10% trifluoroacetic acid (Sigma) and HPLC water. The target plate was introduced into the MALDI-TOF MS instrument for analysis. To control for differences in sample loading, matrix quality and MALDI-TOF apparatus performance, the matrix solution was loaded in duplicate onto each MALDI-TOF plate with and without a bacterial test standard (Bruker Bacterial Test Standard, ref: #8255343).

#### Spectra analysis and reference database creation

A Microflex MALDI-TOF Mass Spectrometer (Bruker Daltonics, Germany) was used to generate MS ranging from 2 to 20 kDa. Spectra were acquired in positive linear mode at a laser frequency of 50 Hz. The acceleration voltage was 20 kV, and the extraction delay time was 200 ns. Each spectrum corresponded to ions obtained from 240 laser shots performed in six regions of the same spot and automatically acquired using the AutoXecute method of the flexControl v2.4 software (Bruker Daltonics). The reproducibility of MALDI-TOF MS spectra from all compartments of mosquitoes infected or not infected with filariae was evaluated by comparing the average spectra obtained from the four spectra of each sample tested using Flex analysis and ClinProTools 2.2 software (Bruker Daltonics). The specificity of MALDI-TOF MS spectra according to the filariae species with which the mosquitoes were infected was also analyzed using the Flex analysis and ClinProTools 2.2 software (Bruker Daltonics). To create a MS database, reference spectra (MSP, Main Spectrum Profile) were created by combining the results of the spectra from at least two individual mosquitoes per compartment using the automated function on the MALDI-Biotyper software v3.0. (Bruker Daltonics). Thus, the MS of a total of 47 specimens was used to build the database. These reference spectra were added to the homemade database containing 915 MS from eight arthropod families, including MS profiles from 30 adult mosquito species [27].

#### Blind tests, sensitivity and specificity estimation

**Fig 1:**
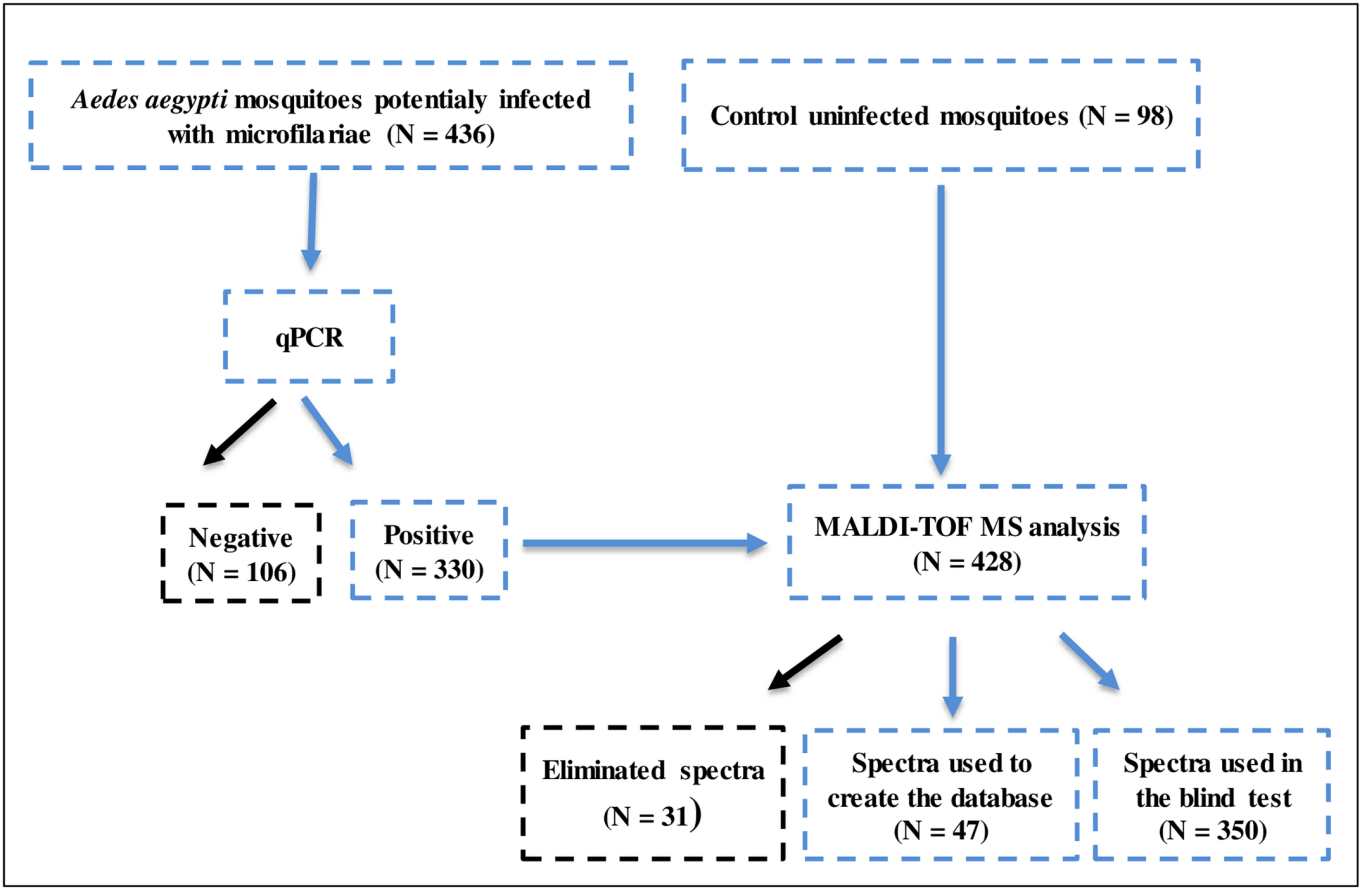
Schematic representation of the molecular and MS analysis performed in this study.

To test the performance of MALDI-TOF MS in screening mosquitoes infected or not infected with microfilariae, a blind test was carried out using 1,400 MS spectra obtained from 350 mosquitoes infected or not infected with filariae (Fig 1). All spectra considered poor quality (i.e. low intensity), as well as spectra that were introduced in the reference database, were excluded from the test. Blind tests were performed using the MALDI-Biotyper software v3.0. tool (Bruker Daltonics). The level of significance was determined using the LSV, which ranged from 0 to 3. The log score value given by the MALDI-Biotyper software v.3.3. corresponds to a match between the query’s MS and reference spectra’s signal intensities. A sample was considered correctly and significantly identified when the queried spectrum obtained an LSV ≥ 1.8 [21].

Sensitivity (Se) and specificity (Sp) were calculated using formulae reported in the literature (Altman et Bland, 1994) [28]:

$$Sensitivity\;(\%)=100\;x\;\frac{TP}{TP+FN}$$

$$Specificity\;(\%)=100\;x\;\frac{TN}{TN+FP}$$

TP: true positives, FN: false negatives, TN: true negatives, FP: false positives

## Results

### Infection rates using qPCR

Infection rates for each group of *Ae*. *aegypti* tested by qPCR were as follows: in Group 1 (legs to be tested by MALDI-TOF MS), *D*. *immitis*, *B*. *malayi* and *B*. *pahangi* DNA were respectively detected in 77.3% (58/75), 90% (54/60) and 83.5% (66/79) of mosquitoes (Table 1). Infection rates for Group 2 (Thorax to be tested by MALDI-TOF MS), the infection rates were 73.9% (17/23), 95.4% (42/44) and 80% (12/15) for *D*. *immitis*, *B*. *malayi* and *B*. *pahangi*, respectively. As for Group 3 (thorax-head to be tested by MALDI-TOF MS), were 28.6% (18/63), 62.5% (10/16) and 79.16% (19/24) for *D*. *immitis*, *B*. *malayi* and *B*. *pahangi*, respectively. Lastly, the infection rates for Group 4 (head to be tested by MALDI-TOF MS), were 95.6% (22/23) and 85.7% (12/14) *B*. *malayi* and *B*. *pahangi*, respectively (*D*. *immitis* was not tested by qPCR for this group because of a lack of samples) (Table 1).

### Spectral analysis

A total of 428 body parts of *Ae*. *aegypti* mosquitoes were submitted for MALDI-TOF MS analysis. First, the MS spectra were assessed visually by comparing the average spectra (MSP Main Spectrum Profile) obtained from the four spectra of each sample tested using the flexAnalysis v3.3 and ClinProTools v2.2 software (Bruker Daltonics). Inadequate spectra (i.e. MS with low quality) were excluded from the study. For example, all samples providing MS of which the most intense peaks were less than 2000 a.u. or with no detected spectra were systematically excluded. Based on these criteria, a total of 31 MS were excluded from the study. Next, spectra with a good reproducibility of at least two specimens per compartment (control uninfected and filariae infected mosquitoes) were randomly selected and loaded in MALDI-Biotyper 3.0 software to create a reference database. Thus, a total of 47 mosquito body parts were used to create this reference database. They are allocated as follows: 21 legs (5 control, 5 infected with *D*. *immitis*, 5 infected with *B*. *malayi* and 6 infected with *B*. *pahangi*), 9 thorax (2 control, 2 infected with *D*. *immitis*, 3 infected with *B*. *malayi* and 2 infected with *B*. *pahangi*), 10 thorax-head (3 control, 2 infected with *D*. *immitis*, 2 infected with *B*. *malayi* and 3 infected with *B*. *pahangi*) and 7 head (3 control, 2 infected with *B*. *malayi* and 2 infected with *B*. *pahangi*).

**Fig 2:**
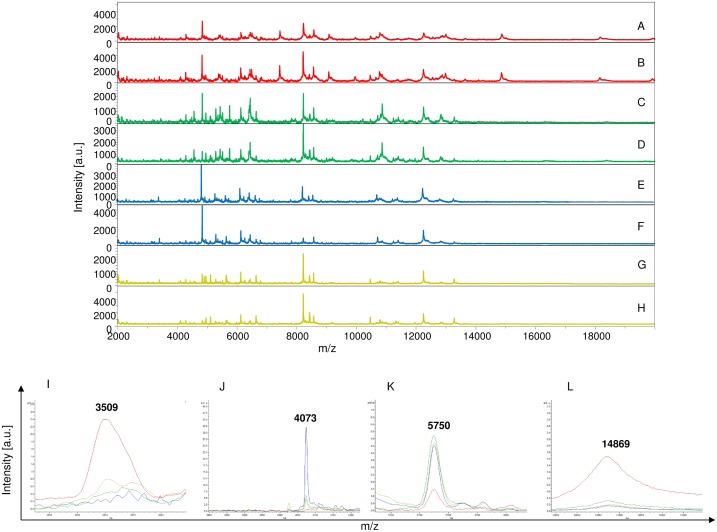
Comparison of MALDI-TOF MS spectra from legs of *Aedes aegypti* infected or not by filariae. Spectra of control *Ae*. *aegypti* not exposed to filariae (A, B) or infected with *D*. *immitis* (C, D) or *B*. *malayi* (E, F) or *B*. *pahangi* (G, H). The filariae infectious status for each specimen was controlled by qPCR. Some distinct protein masses detected with ClinProTools software are represented (I, J, K, and L). a.u., arbitrary units; m/z, mass-to-charge ratio.

**Fig 3:**
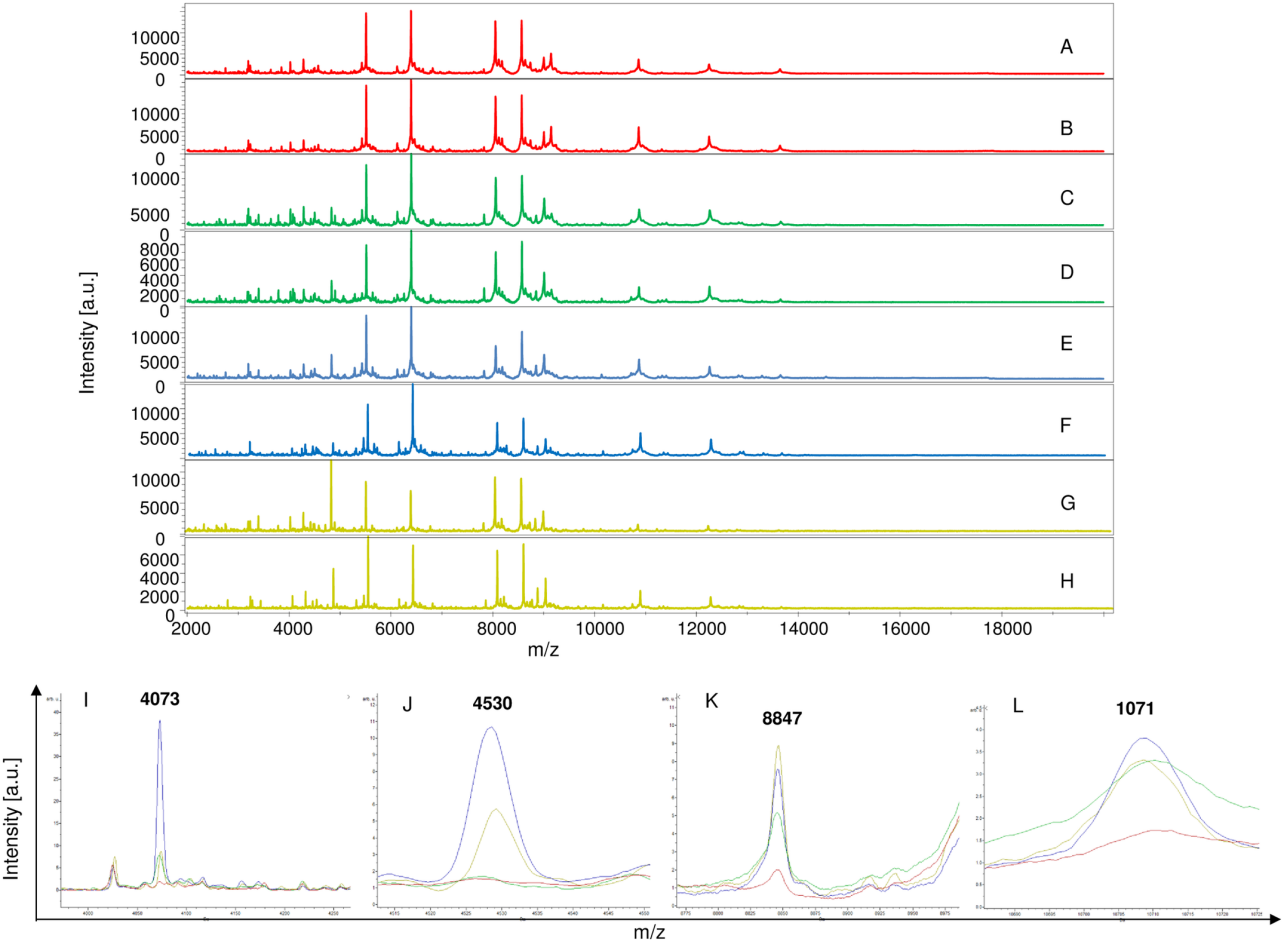
Comparison of MALDI-TOF MS spectra from thorax of *Aedes aegypti* infected or not by filariae. Spectra of control uninfected *Ae*. *aegypti* (A, B); infected with *D*. *immitis* (C, D) or *B*. *malayi* (E, F) or *B*. *pahangi* (G, H). Some distinct protein masses generated with ClinProTools are represented (I, J, K, and L). a.u., arbitrary units; m/z, mass-to-charge ratio.

**Fig 4:**
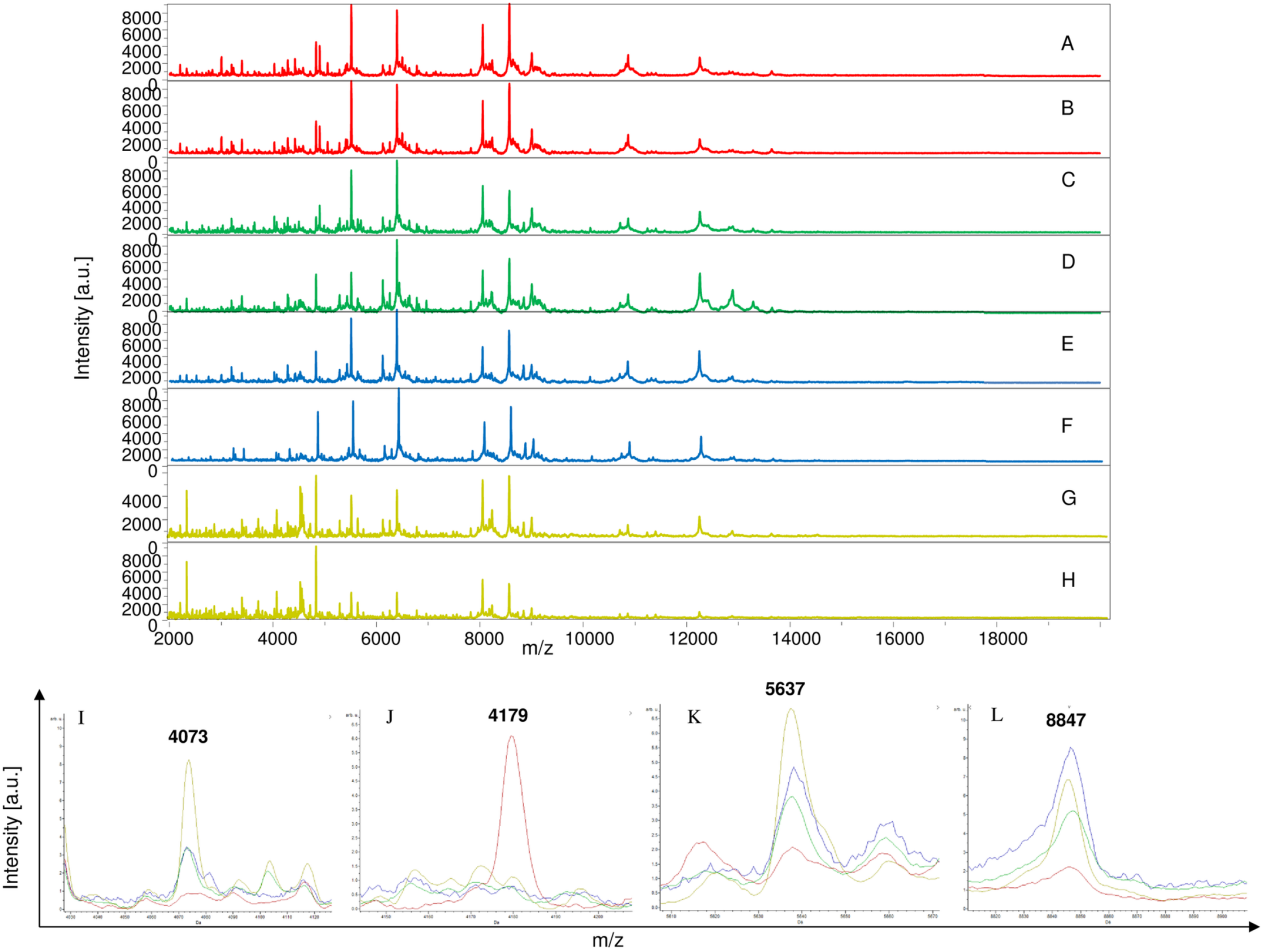
Comparison of MALDI-TOF MS spectra from thorax-head of *Aedes aegypti* infected or not by filariae. Spectra of control uninfected *Ae*. *aegypti* (A, B); infected with *D*. *immitis* (C, D) or *B*. *malayi* (E, F) or *B*. *pahangi* (G, H). Somme distinct protein masses generated with ClinProTools are represented (I, J, K, and L). a.u., arbitrary units; m/z, mass-to-charge ratio.

**Fig 5:**
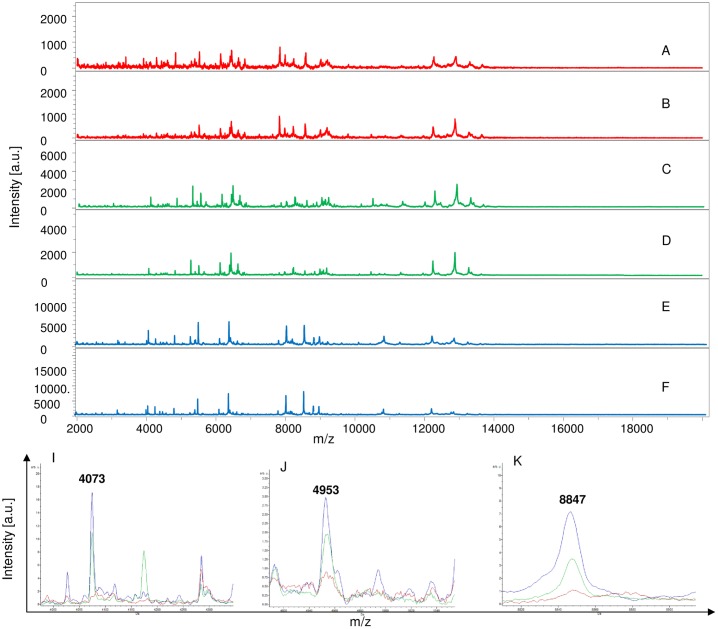
Comparison of MALDI-TOF MS spectra from heads of *Aedes aegypti* infected or not by filariae. Spectra of control uninfected *Ae*. *aegypti* (A, B); infected with *B*. *malayi* (C, D) or *B*. *pahangi* (E, F). Some distinct protein masses generated with ClinProTools are represented (I, J and K). a.u., arbitrary units; m/z, mass-to-charge ratio.

The remaining MS (350 mosquito parts) were blind tested against the database. Visual inspection of spectral profiles obtained from different compartments showed consistent and reproducible spectra between specimens according to the compartments, namely legs (Fig 2), thorax (Fig 3), thorax-head (Fig 4) and head (Fig 5), and the infectious status. Spectra alignment using Flex analysis software confirmed reproducibility but also revealed changes in the MS pattern according to the infectious status, with mass peaks present or absent between infected and uninfected mosquitoes.

### Peak masses distinguishing non-infected and infected mosquitoes

In total, 37 discriminating peak masses ranging from 2062 to 14869 Da were identified (Table 2), of which 17, 11, 12 and 7 peak masses were for legs, thorax, thorax-head and head spectra respectively. For Group 1 (legs), regardless of the filariae species with which mosquitoes were infected, spectral profile analysis showed that there were at least two protein peaks (3509 and 14869 Da) only present in spectra obtained from control mosquitoes compared to the infected ones (Fig 2), while three peak masses (2062 and 4073 and 8847 Da) were exclusively present in infected mosquitoes (Table 2). For Group 2 (thorax), three protein peaks (4073, 8847 and 1071 Da) were present in the infected mosquitoes compared to the non-infected ones (Fig 3). As for Group 3 (thorax-head), six protein peaks (4073, 5637 and 8847 Da) and (2759, 4179 and 6498 Da) were only found in infected and control uninfected mosquitoes, respectively (Fig 4 and Table 2). Finally, for Group 4 (head), two protein peaks (4073 and 8847 Da) were found only in infected specimens compared to uninfected ones. It is important to note that of the 37 peak masses, two (4073 and 8847 Da) were observed in all groups of filariae infected mosquitoes (regardless of species) compared with uninfected ones (Table 2).

**Table 2:** Peak masses distinguishing uninfected and filariae-infected *Aedes aegypti* mosquitoes according the compartment, based on the Genetic Algorithm model analysis of ClinProTools.

Peak masses (Da)	Group 1: legs				Group 2: thorax				Group 3: thorax-head				Group 4: head			
	Ctrl	D. I	B. M	B. P	Ctrl	D. I	B. M	B. P	Ctrl	D. I	B. M	B. P	Ctrl	D. I	B. M	B. P
2062	-	+++	+	++												
2142	++	+	+	+												
2329					+	+	+++	+++								
2759									+++	-	-	-				
2828													+++	/	++	+
3001					+	+	+	+++	++	++	+	++				
3028									+	++	++	++				
3253					+++	+	+	+								
3398	+	+	++	++												
3509	+++	-	-	-												
3514	+	+	+	+++												
3527					+	+	-	+								
3640					+++	+++	+	+++								
3755					++	++	-	++								
4025	+	+	+	++												
4073	-	+++	+++	+++	-	+++	+++	+++	-	++	++	+++	-	/	+++	+++
4179									+++	-	-	-				
4530					-	-	+++	+++								
4953													+	/	++	++
5056									+++	+	-	-				
5135									+++	+	+	+				
5284					+	++	++	++	+	++	++	++	+	/	+++	+++
5290	+	+	+++	+++												
5637									-	++	++	++				
5639	+	+	++	++												
5750	+	+++	+++	+++												
6126	+	+	+++	+++												
6498									+++	-	-	-				
6781	+	+	+++	+++												
7826	+	+	+++	+++												
8433	+	+	++	+												
8847	-	++	++	++	-	+++	+++	+++	-	+++	+++	+++	-	/	+++	+++
9799													++	/	-	-
1071	+	++	++	++	-	++	++	++								
11955													-	/	+++	+++
12253	+	+	++	++												
14869	+++	-	-	-												
Total discriminating peak	**17**				**11**				**12**				**7**			

Ctrl: Control (non-infected), D. I: *Dirofilaria immitis*, B. M: *Brugia malayi*, B. P: *Brugia pahangi*.

Discriminating peak masses can be present in all filarioid-infected specimens but overexpressed for one species more than others. For example, the 5290, 6126, 6781, 7827 Da peaks are intensely expressed in mosquitoes’ legs (Group 1) infected with *B*. *malayi* and *B*. *pahangi* compared to the *D*. *immitis* infected specimens (Table 2). Concerning Group 2, the 2329 Da peak was intensely expressed in the mosquitoes infected with *B*. *malayi* and *B*. *pahangi* compared to those infected with *D*. *immitis*. As for Group 3, the 3001 Da peak was more noticeable in the mosquitoes infected with *D*. *immitis* and *B*. *pahangi* compared to those infected with *B*. *malayi*. Finally, for Group 4, one peak (2828 Da) was more expressed in mosquitoes infected with *B*. *malayi* compared to those infected with *B*. *pahangi*.

### Blind tests

The specificity, estimated using control uninfected mosquitoes, varied slightly depending on tested compartment. It was 85.1% for leg, 76.9% for thorax, 94.1% for thorax-head and 80% for head analysis. In addition, the blind test showed correct identification rates for infected specimens varying according to the compartment tested. The sensitivity was 82.9% for legs, 60% for thorax and 86.6% for thorax-heads infected with *D*. *immitis* (Table 3). It was 61.6% for legs, 65.7% for thorax, 71.4% for thorax-head and 84.2% for heads infected with *B*. *malayi*. It was 75.4% for legs, 70% for thorax, 68.7% for thorax-heads and 70% for heads infected with *B*. *pahangi*.

**Table 3:** Result of the blind tests against the MALDI-TOF MS reference database according the compartment and infectious status of mosquitoes.

		Number of specimens	Number of specimens excluded from analysis	Specimens integrated in the database (N)	Specimens used for the blind test (N)	High LSVs obtained from blind test against Database		Correct classification (%) (Specificity and sensitivity)	
						Correct classification	Incorrect classification		
**Legs**	**Control mosquitoes**	**40**	**8**	**5**	**27**	**23**	**4** (2 DI, 1 BM)	**85.18**	
	**Mosquitoes infected with *D*. *immitis***	**58**	**6**	**5**	**47**	**39**	**8** (3 DI, 4 BP, 1 Neg)	**82.97**	97.87*
	**Mosquitoes infected with *B*. *malayi***	**54**	**5**	**5**	**44**	**27**	**17** (12 BP, 3 DI, 2 Neg)	**61.63**	95.45*
	**Mosquitoes infected with *B*. *pahangi***	**66**	**3**	**6**	**57**	**43**	**14** (BM)	**75.43**	100*
	**Total**	**218**	**22**	**21**	**175**	**132**	**43**	/	
**Thorax**	**Control mosquitoes**	**15**	**0**	**2**	**13**	**10**	**3** (2 Thx-Hd DI, 1 Thx BP)	**76.92**	
	**Mosquitoes infected with *D*. *immitis***	**17**	**0**	**2**	**15**	**9**	**6** (3 Thx-Hd DI, 3 Thx-BM)	**60**	100*
	**Mosquitoes infected with *B*. *malayi***	**42**	**1**	**3**	**38**	**25**	**13** (4 Thx-Hd DI, 7 Thx-Hd BM, 1 Thx DI, 1 Neg))	**65.78**	97.36*
	**Mosquitoes infected with *B*. *pahangi***	**12**	**0**	**2**	**10**	**7**	**3** (1 Thx-Hd DI, 2 Thx-Hd BM)	**70**	100*
	**Total**	**86**	**1**	**9**	**76**	**51**	**25**	/	
**Thorax-head**	**Control mosquitoes**	**22**	**2**	**3**	**17**	**16**	**1** (Thx-Hd DI)	**94.11**	
	**Mosquitoes infected with *D*. *immitis***	**18**	**1**	**2**	**15**	**13**	**2** (Thx-Hd BM)	**86.66**	100*
	**Mosquitoes infected with *B*. *malayi***	**10**	**1**	**2**	**7**	**5**	**2** (1 Thx-Hd DI, 1 Hd-BP)	**71.42**	100*
	**Mosquitoes infected with *B*. *pahangi***	**19**	**0**	**3**	**16**	**11**	**5** (1 Thx-Cl, 2 Thx-Hd-DI, 2 Thx-Hd-BM)	**68.75**	100*
	**Total**	**69**	**4**	**10**	**55**	**45**	**10**	/	
**Head**	**Control mosquitoes**	**21**	**3**	**3**	**15**	**12**	**3** (Thx-Hd DI)	**80**	
	**Mosquitoes infected with *B*. *malayi***	**22**	**1**	**2**	**19**	**16**	**3 (1** Thx-Hd DI, **2** Thx-Hd BM)	**84.21**	100*
	**Mosquitoes infected with *B*. *pahangi***	**12**	**0**	**2**	**10**	**7**	**2** (Hd BM, 1 Neg)	**70**	90*
	**Total**	**55**	**4**	**7**	**44**	**35**	**9**	/	
**Total**		**428**	**31**	**47**	**350**	**263**	**87**	/	

DI: *Dirofilaria immitis*, BM: *Brugia malayi*, BP: *Brugia pahangi*, Thx: thorax, Cl: control, Hd: head, Neg: negative.

The asterisk (*) indicates the sensitivity of the MALDI-TOF MS without taking into account the filariae species of which mosquitoes were infected.

## Discussion

This is the first study conducted on using MALDI-TOF MS to detect filariae in mosquitoes. Here, the ability of the MALDI-TOF MS to detect filariae in mosquitoes was evaluated using qPCR as a “gold standard”/reference. The reliability of nucleic acid amplification techniques for filariae detection in vectors has been addressed in a number of studies [25,26,29]. These studies showed that these PCR assays had high sensitivity and specificity toward the detection of the filariae in mosquitoes.

In a recent study [22] it was reported that MALDI-TOF MS can correctly screen (100% of specificity and 92% and sensitivity) mosquitoes infected or not infected with *Plasmodium berghei* parasites using head and thorax as the target part. Here, we investigated whether the MALDI-TOF MS tool could detect changes in the protein profiles of non-infected and filariae-infected *Ae*. *aegypti* mosquitoes, in other words generate a profile reflecting the infectious status. We were also interested in assessing which mosquito compartment was appropriate for determining infectious status using MALDI-TOF MS.

It is worth noting that after ingestion by the mosquito, *Dirofilaria* spp. microfilariae remain in the midgut for approximately 24 h. Subsequently, they migrate into the large cells of the malpighian tubules [6]. After two molts (L2, L3) the filariae perforate the distal ends of the tubules and migrate via the haemocoel to the head of the mosquito on the 15^th^ to 17^th^ day [30,31]. For *Brugia* spp. development in mosquito, after ingestion, the microfilariae lose their sheaths and perforate the wall of the proventriculus and cardiac portion of the midgut to reach the thoracic muscles [32]. At this level, the microfilariae develop into first-stage larvae (L1) and subsequently into third-stage larvae (L3) within 8 to 10 days after the infecting blood meal [32,33]. Subsequently, the L3 larvae migrate through the hemocoel to the mosquito's prosbocis on the within 14 to 20 days. A small minority of larvae may stay in the haemocoele or enter some other thoracic structure in which they stay without signs of development [33].

Previous studies showed that changes occur in the mosquitoes' hemolymph as a result of infection by microorganisms [34,34–36] and this can provide a useful approach for examining changes in hemolymph proteins after infection by parasites [36]. It is acknowledged that these proteins may play important roles in the relationship between mosquitoes and the viral, protozoal and nematode pathogens they transmit [36]. In their study, Paskewitz et al. (2005) focused on evaluating changes in the protein profiles in the hemolymph of *Anopheles gambiae* following bacterial (*Escherichia coli*) inoculation, identifying 26 hemolymph proteins that belong to families linked to immunity, lipid transport, and iron regulation in insects [36]. Shi et al. (2004) reported two bacterial infection-related proteins in *An*. *gambiae* hemolymph using 2D SDS PAGE analysis. These two mosquito proteins are involved in immunity because they appear early in the hemolymph following mosquito exposure to bacterial infection, but not to other treatments that cause damage to the mosquito's body wall [37]. In their study, Brenda et al. (1990) showed that there is an increase in biosynthesis of the 84-kDa polypeptide in the hemolymph of *Ae*. *aegypti* mosquitoes inoculated with *D*. *immitis* microfilariae compared with those from saline-inoculated and uninoculated controls [38]. According to these authors, greater synthetization of this protein in *D*. *immitis*-inoculated mosquitoes may reflect the production of melanotic material necessary for the encapsulation reactions against microfilariae parasites [38]. All these changes occurring at the hemolymph level represent one of the reasons why we tested different parts of mosquitoes, including legs.

Here, the infectious status of each mosquito was validated by means of a high sensitivity molecular tool. It has been demonstrated by dissecting mosquitoes that filariae, especially L3 stage larvae, are found in the abdominal hemocoel 15 days after infection [39]. This validates our analysis approach, in which we have tested the abdomen by qPCR in each group to detect filariae DNA, especially for the group in which head and thorax were tested by MALDI-TOF MS as a whole part. Nevertheless, for this group (Group 3), a low infection rate (28.5%) for *D*. *immitis* was obtained, compared to Group 1 and Group 2 in which the infection rates were 77.3% and 73.9% respectively. This result can be explained by the low density (or absence) of the filariae present in the abdomen of the mosquito after migration of the L3 larvae to the head two weeks after the infecting blood meal.

A comparison of spectra profiles for control and infected mosquitoes using ClinProTools showed a set of 37 biomarker masses that distinguish mosquitoes according to their infectious status as well as the filariae species with which the mosquito was infected. Of these peak masses, some are present only in infected specimens. It may be inferred that these proteins correspond to filariae proteins circulation or to the immune-induced proteins of the mosquitoes following infection as previously reported in mosquitoes and *Drosophila* fruit flies challenged with bacteria [36] [40]. Furthermore, we have noted that some discriminating peaks are detected in the uninfected control mosquitoes and are down-regulated or squarely suppressed in the infected specimens. This agrees with published literature in which it has been reported that certain genes coding for proteins involved in innate immunity are down-regulated after bacterial or malaria challenges of *Anopheles gambiae* mosquitoes [41]. The performance of MALDI-TOF MS for filariae detection in different *Ae*. *aegypti* mosquitoes’ compartments was based on the blind test following the database’s creation. The obtained results generally presented specificity and sensitivity rates ranging from 76.9% to 94.1%, and from 60% to 86.6% respectively, according to the target compartment. For legs (Group 1), the specificity is 85.1% while the sensitivity is 82.9%, 61.3% and 75.4% for specimens infected with *D*. *immits*, *B*. *malayi* and *B*. *pahangi* respectively. These values were closer than those reported in a previous study (93.7% of specificity and 88.9% of sensitivity) in which another pathogen (*Borrelia crocidurae*) was detected in the legs of *Ornithodoros sonrai* ticks using MALDI-TOF MS [20]. For thorax (Group 2), the specificity is 76.9% while the sensitivity is 60%, 65.7% and 70% for specimens infected with *D*. *immitis*, *B*. *malayi* and *B*. *pahangi* respectively. The best specificity and sensitivity results were obtained from the thorax-head compartment (Group 3) with values of 94.1% and 86.6%, 71.4% and 68.7% for control uninfected mosquitoes and specimens infected with *D*. *immitis*, *B*. *malayi* and *B*. *pahangi* respectively. In their study, Laroche et al. (2017) had better results (100% of specificity and 92.8% of sensitivity) testing the thorax-head by MALDI-TOF MS to screen *Anopheles stephensi* mosquitoes infected or not infected with *Plasmodium berghei* parasites [22]. Lastly, the head (Group 4) generated a specificity of 80% and a sensitivity of 84.2% and 70% for control uninfected mosquitoes and specimens infected with *B*. *malayi* and *B*. *pahangi* respectively. All these values of specificity and sensitivity can be considered good taking into account some limitations of the MALDI-TOF MS such as the relative low resolution and limited sensitivity for larger masses (MS superior to 20 kDa) [13]. This limitation may make this tool unable to detect all proteins that can differentiate the filariae species for which mosquitoes are tested. However, another promising technique can be used in combination with MALDI-TOF MS. This method, known as peptide mass fingerprinting or shotgun mass mapping, involves the proteolytic hydrolysis of the sample prior to MALDI-TOF MS reference database creation or interrogation [13,42]. It is based on the comparison of peptide MS spectra. The advantages of shotgun mass mapping are greater resolution in the lower mass range (*i*.*e*. from 500 to 4000 Da) and the ability to obtain peptide sequence information by analyzing the more stringent peptides with tandem mass spectrometry [13]. It is worth noting that the application of this technique in medical entomology has been successfully initiated by Uhlmann et al. (2014), by determining the identity of 28 peptide peaks of Culicoides in which the mass ranged from 1.1 to 3.1 kDa [42].

This study demonstrated the potential of MALDI-TOF MS as a promising tool for screening *Aedes aegypti* mosquitoes as being non-infected or filariae-infected. For large scale studies, this technique can be applied to screen mosquitoes (infected/not infected) and then other tools can be used, such as PCR for pathogen species identification. Moreover, it is recognized that the MALDI-TOF MS-based approaches provides cheaper and faster method for routine microbial species identification than conventional phenotypic and 16S molecular sequencing identification methods, with equal or better accuracy [18,43–45]. In a study conducted by Dhiman N et al. (2011) [46] the authors reported a reagent cost of $0.50 and an average hands-on-time of 5.1 min per isolate for yeast identification. In their study, Cherkaoui et al. (2010) [47] reported that of a total of 720 isolates belonging to different bacterial species, the average cost of conventional and MALDI-TOF MS identifications was approximately $10 and $0.50 per isolate respectively. In addition, the estimated timeliness of conventional and MALDI-TOF MS methods was 24 h and 5 min per isolate, respectively. In a cost-benefit study published in 2015, showed that out of 21,930 isolates composed of commonly isolated organisms (*e*.*g*., bacteria and yeast) the total costs with traditional methods, including reagent, technologist time, and maintenance agreement contracts, were determined to be $6.50 per isolate reported, compared to $3.14 for with MALDI-TOF MS [44]. It is noteworthy that for 16S molecular sequencing, reagent costs are 5–10 times higher than of MALDI-TOF MS [44]. In addition, the cost of the instrument and software ($150,000) is comparable to that for DNA-sequencing platforms [46]. This suggests that, once the MALDI Biotyper machine is purchased, the analyzing cost per sample remains much lower by MALDI-TOF MS than by molecular biology. This implies that in the coming years, MALDI-TOF MS will be a routine tool in monitoring and managing human and animal vector-borne diseases (*e*.*g*. filariosis). Furthermore, we recommend that other studies be conducted using other species of mosquitoes challenged with different filarioid species to create a large database and consolidate the results obtained in this scope of research. Additionally, the characterization of the proteins (i.e. amino acid composition and sequence) from discriminating peaks will precise the protein candidates involved in MS profile changes following nematode infection.

## References

[1] Benelli G, Jeffries CL, Walker T (2016) Biological Control of Mosquito Vectors: Past, Present, and Future. Insects 7 doi: 10.3390/insects7040052 27706105 10.3390/insects7040052 PMC5198200

[2] Pavela R, Benelli G (2016) Ethnobotanical knowledge on botanical repellents employed in the African region against mosquito vectors—A review. Exp Parasitol 167: 103–108. doi: 10.1016/j.exppara.2016.05.010 27260568 10.1016/j.exppara.2016.05.010

[3] Becker N (2008) Influence of climate change on mosquito development and mosquito-borne diseases in Europe. Parasitol Res 103 Suppl 1: S19–S28. doi: 10.1007/s00436-008-1210-2 19030883 10.1007/s00436-008-1210-2

[4] Benelli G, Mehlhorn H (2016) Declining malaria, rising of dengue and Zika virus: insights for mosquito vector control. Parasitol Res 115: 1747–1754. doi: 10.1007/s00436-016-4971-z 26932263 10.1007/s00436-016-4971-z

[5] Dieme C, Bechah Y, Socolovschi C, Audoly G, Berenger JM, Faye O, Raoult D, Parola P (2015) Transmission potential of *Rickettsia felis* infection by *Anopheles gambiae* mosquitoes. Proc Natl Acad Sci U S A 112: 8088–8093. doi: 10.1073/pnas.1413835112 26056256 10.1073/pnas.1413835112 PMC4491796

[6] McCall JW, Genchi C, Kramer LH, Guerrero J, Venco L (2008) Heartworm disease in animals and humans. Adv Parasitol 66: 193–285. doi: 10.1016/S0065-308X(08)00204-2 18486691 10.1016/S0065-308X(08)00204-2

[7] Dantas-Torres F, Otranto D (2013) Dirofilariosis in the Americas: a more virulent *Dirofilaria immitis*? Parasit Vectors 6: 288 doi: 10.1186/1756-3305-6-288 24274042 10.1186/1756-3305-6-288 PMC3851770

[8] Simon F, Siles-Lucas M, Morchon R, Gonzalez-Miguel J, Mellado I, Carreton E, Montoya-Alonso JA (2012) Human and animal dirofilariasis: the emergence of a zoonotic mosaic. Clin Microbiol Rev 25: 507–544. doi: 10.1128/CMR.00012-12 22763636 10.1128/CMR.00012-12 PMC3416488

[9] Simonsen PE, Mwakitalu ME (2013) Urban lymphatic filariasis. Parasitol Res 112: 35–44. doi: 10.1007/s00436-012-3226-x 23239094 10.1007/s00436-012-3226-x PMC3536942

[10] World Health Organisation (WHO). Lymphatic filariasis. 2016; http://www.who.int/lymphatic_filariasis/disease/en/

[11] Tan LH, Fong MY, Mahmud R, Muslim A, Lau YL, Kamarulzaman A (2011) Zoonotic *Brugia pahangi* filariasis in a suburbia of Kuala Lumpur City, Malaysia. Parasitol Int 60: 111–113. doi: 10.1016/j.parint.2010.09.010 20951228 10.1016/j.parint.2010.09.010

[12] Bockarie MJ, Pedersen EM, White GB, Michael E (2009) Role of vector control in the global program to eliminate lymphatic filariasis. Annu Rev Entomol 54: 469–487. doi: 10.1146/annurev.ento.54.110807.090626 18798707 10.1146/annurev.ento.54.110807.090626

[13] Yssouf A, Almeras L, Raoult D, Parola P (2016) Emerging tools for identification of arthropod vectors. Future Microbiol 11: 549–566. doi: 10.2217/fmb.16.5 27070074 10.2217/fmb.16.5

[14] Plichart C, Sechan Y, Davies N, Legrand AM (2006) PCR and dissection as tools to monitor filarial infection of *Aedes polynesiensis* mosquitoes in French Polynesia. Filaria J 5: 2 doi: 10.1186/1475-2883-5-2 16504131 10.1186/1475-2883-5-2 PMC1403774

[15] Licitra B, Chambers EW, Kelly R, Burkot TR (2010) Detection of *Dirofilaria immitis* (Nematoda: Filarioidea) by polymerase chain reaction in *Aedes albopictus*, *Anopheles punctipennis*, and *Anopheles crucians* (Diptera: Culicidae) from Georgia, USA. J Med Entomol 47: 634–638. 20695279 10.1093/jmedent/47.4.634 PMC7027258

[16] Moustafa MA, Salamah MM, Thabet HS, Tawfik RA, Mehrez MM, Hamdy DM (2017) Molecular xenomonitoring (MX) and transmission assessment survey (TAS) of lymphatic filariasis elimination in two villages, Menoufyia Governorate, Egypt. Eur J Clin Microbiol Infect Dis. doi: 10.1007/s10096-017-2901-3 28155014 10.1007/s10096-017-2901-3

[17] Lau CL, Won KY, Lammie PJ, Graves PM (2016) Lymphatic Filariasis Elimination in American Samoa: Evaluation of Molecular Xenomonitoring as a Surveillance Tool in the Endgame. PLoS Negl Trop Dis 10: e0005108 doi: 10.1371/journal.pntd.0005108 27802280 10.1371/journal.pntd.0005108 PMC5089733

[18] Seng P, Drancourt M, Gouriet F, La SB, Fournier PE, Rolain JM, Raoult D (2009) Ongoing revolution in bacteriology: routine identification of bacteria by matrix-assisted laser desorption ionization time-of-flight mass spectrometry. Clin Infect Dis 49: 543–551. doi: 10.1086/600885 19583519 10.1086/600885

[19] Chalupova J, Raus M, Sedlarova M, Sebela M (2014) Identification of fungal microorganisms by MALDI-TOF mass spectrometry. Biotechnol Adv 32: 230–241. doi: 10.1016/j.biotechadv.2013.11.002 24211254 10.1016/j.biotechadv.2013.11.002

[20] Fotso FA, Mediannikov O, Diatta G, Almeras L, Flaudrops C, Parola P, Drancourt M (2014) MALDI-TOF mass spectrometry detection of pathogens in vectors: the *Borrelia crocidurae*/*Ornithodoros sonrai* paradigm. PLoS Negl Trop Dis 8: e2984 doi: 10.1371/journal.pntd.0002984 25058611 10.1371/journal.pntd.0002984 PMC4109908

[21] Yssouf A, Almeras L, Terras J, Socolovschi C, Raoult D, Parola P (2015) Detection of *Rickettsia* spp in ticks by MALDI-TOF MS. PLoS Negl Trop Dis 9: e0003473 doi: 10.1371/journal.pntd.0003473 25659152 10.1371/journal.pntd.0003473 PMC4319929

[22] Laroche M, Almeras L, Pecchi E, Bechah Y, Raoult D, Viola A, Parola P (2017) MALDI-TOF MS as an innovative tool for detection of *Plasmodium* parasites in *Anopheles* mosquitoes. Malar J 16: 5 doi: 10.1186/s12936-016-1657-z 28049524 10.1186/s12936-016-1657-z PMC5209920

[23] Chandrashekar R, Beall MJ, Saucier J, O'Connor T, McCall JW, McCall SD (2014) Experimental *Dirofilaria immitis* infection in dogs: effects of doxycycline and Advantage Multi(R) administration on immature adult parasites. Vet Parasitol 206: 93–98. doi: 10.1016/j.vetpar.2014.08.011 25218886 10.1016/j.vetpar.2014.08.011

[24] Cosgrove JB, Wood RJ, Petric D, Evans DT, Abbott RH (1994) A convenient mosquito membrane feeding system. J Am Mosq Control Assoc 10: 434–436. 7807091

[25] Tahir D, Bittar F, Barre-Cardi H, Sow D, Dahmani M, Mediannikov O, Raoult D, Davoust B, Parola P (2017) Molecular survey of *Dirofilaria immitis* and *Dirofilaria repens* by new real-time TaqMan(R) PCR assay in dogs and mosquitoes (Diptera: Culicidae) in Corsica (France). Vet Parasitol 235: 1–7. doi: 10.1016/j.vetpar.2017.01.002 28215858 10.1016/j.vetpar.2017.01.002

[26] Rao RU, Weil GJ, Fischer K, Supali T, Fischer P (2006) Detection of *Brugia parasite* DNA in human blood by real-time PCR. J Clin Microbiol 44: 3887–3893. doi: 10.1128/JCM.00969-06 16957038 10.1128/JCM.00969-06 PMC1698366

[27] Nebbak A, Willcox AC, Bitam I, Raoult D, Parola P, Almeras L (2016) Standardization of sample homogenization for mosquito identification using an innovative proteomic tool based on protein profiling. Proteomics 16: 3148–3160. doi: 10.1002/pmic.201600287 27862981 10.1002/pmic.201600287

[28] Altman DG, Bland JM (1994) Diagnostic tests. 1: Sensitivity and specificity. BMJ 308: 1552 8019315 10.1136/bmj.308.6943.1552 PMC2540489

[29] Albonico F, Loiacono M, Gioia G, Genchi C, Genchi M, Mortarino M (2014) Rapid differentiation of *Dirofilaria immitis* and *Dirofilaria repens* in canine peripheral blood by real-time PCR coupled to high resolution melting analysis. Vet Parasitol 200: 128–132. doi: 10.1016/j.vetpar.2013.11.027 24360646 10.1016/j.vetpar.2013.11.027

[30] TAYLOR AE (1960) The development of *Dirofilaria immitis* in the mosquito *Aedes aegypti*. J Helminthol 34: 27–38. 13775556 10.1017/s0022149x00020307

[31] Serrao ML, Labarthe N, Lourenco-de-Oliveira R (2001) Vectorial competence of *Aedes aegypti* (Linnaeus 1762) Rio de Janeiro strain, to *Dirofilaria immitis* (Leidy 1856). Mem Inst Oswaldo Cruz 96: 593–598. 11500754 10.1590/s0074-02762001000500001

[32] Laurence BR, Simpson MG (1971) The microfilaria of *Brugia*: a first stage nematode larva. J Helminthol 45: 23–40. 4396455 10.1017/s0022149x00006908

[33] Beckett EB, Macdonald WW (1970) The distribution of larvae of *Brugia malayi* and *Brugia pahangi* in the flight muscle fibres of *Aedes aegypti* and *Mansonia uniformis*. Parasitology 61: 211–218. 4394923 10.1017/s0031182000041032

[34] Mack SR, Samuels S, Vanderberg JP (1979) Hemolymph of *Anopheles stephensi* from uninfected and *Plasmodium berghei*-infected mosquitoes. 2. Free amino acids. J Parasitol 65: 130–136. 376812

[35] Mack SR, Samuels S, Vanderberg JP (1979) Hemolymph of *Anopheles stephensi* from noninfected and *Plasmodium berghei*-infected mosquitoes. 3. Carbohydrates. J Parasitol 65: 217–221. 376818

[36] Paskewitz SM, Shi L (2005) The hemolymph proteome of *Anopheles gambiae*. Insect Biochem Mol Biol 35: 815–824. doi: 10.1016/j.ibmb.2005.03.002 15944078 10.1016/j.ibmb.2005.03.002

[37] Shi L, Paskewitz SM (2004) Identification and molecular characterization of two immune-responsive chitinase-like proteins from *Anopheles gambiae*. Insect Mol Biol 13: 387–398. doi: 10.1111/j.0962-1075.2004.00496.x 15271211 10.1111/j.0962-1075.2004.00496.x

[38] Beerntsen BT, Christensen BM (1990) Dirofilaria immitis: effect on hemolymph polypeptide synthesis in *Aedes aegypti* during melanotic encapsulation reactions against microfilariae. Exp Parasitol 71: 406–414. 2226702 10.1016/0014-4894(90)90066-l

[39] Nayar JK, Knight JW (1999) *Aedes albopictus* (Diptera: Culicidae): an experimental and natural host of *Dirofilaria immitis* (Filarioidea: Onchocercidae) in Florida, U.S.A. J Med Entomol 36: 441–448. 10467770 10.1093/jmedent/36.4.441

[40] Vierstraete E, Cerstiaens A, Baggerman G, Van den Bergh G, De LA, Schoofs L (2003) Proteomics in *Drosophila melanogaster*: first 2D database of larval hemolymph proteins. Biochem Biophys Res Commun 304: 831–838. 12727233 10.1016/s0006-291x(03)00683-1

[41] Dimopoulos G, Christophides GK, Meister S, Schultz J, White KP, Barillas-Mury C, Kafatos FC (2002) Genome expression analysis of *Anopheles gambiae*: responses to injury, bacterial challenge, and malaria infection. Proc Natl Acad Sci U S A 99: 8814–8819. doi: 10.1073/pnas.092274999 12077297 10.1073/pnas.092274999 PMC124381

[42] Uhlmann KR, Gibb S, Kalkhof S, Arroyo-Abad U, Schulz C, Hoffmann B, Stubbins F, Carpenter S, Beer M, von BM, Feltens R (2014) Species determination of Culicoides biting midges via peptide profiling using matrix-assisted laser desorption ionization mass spectrometry. Parasit Vectors 7: 392 doi: 10.1186/1756-3305-7-392 25152308 10.1186/1756-3305-7-392 PMC4158057

[43] Tan KE, Ellis BC, Lee R, Stamper PD, Zhang SX, Carroll KC (2012) Prospective evaluation of a matrix-assisted laser desorption ionization-time of flight mass spectrometry system in a hospital clinical microbiology laboratory for identification of bacteria and yeasts: a bench-by-bench study for assessing the impact on time to identification and cost-effectiveness. J Clin Microbiol 50: 3301–3308. doi: 10.1128/JCM.01405-12 22855510 10.1128/JCM.01405-12 PMC3457442

[44] Tran A, Alby K, Kerr A, Jones M, Gilligan PH (2015) Cost Savings Realized by Implementation of Routine Microbiological Identification by Matrix-Assisted Laser Desorption Ionization-Time of Flight Mass Spectrometry. J Clin Microbiol 53: 2473–2479. doi: 10.1128/JCM.00833-15 25994167 10.1128/JCM.00833-15 PMC4508454

[45] Lagace-Wiens PR, Adam HJ, Karlowsky JA, Nichol KA, Pang PF, Guenther J, Webb AA, Miller C, Alfa MJ (2012) Identification of blood culture isolates directly from positive blood cultures by use of matrix-assisted laser desorption ionization-time of flight mass spectrometry and a commercial extraction system: analysis of performance, cost, and turnaround time. J Clin Microbiol 50: 3324–3328. doi: 10.1128/JCM.01479-12 22875888 10.1128/JCM.01479-12 PMC3457416

[46] Dhiman N, Hall L, Wohlfiel SL, Buckwalter SP, Wengenack NL (2011) Performance and cost analysis of matrix-assisted laser desorption ionization-time of flight mass spectrometry for routine identification of yeast. J Clin Microbiol 49: 1614–1616. doi: 10.1128/JCM.02381-10 21270234 10.1128/JCM.02381-10 PMC3122878

[47] Cherkaoui A, Hibbs J, Emonet S, Tangomo M, Girard M, Francois P, Schrenzel J (2010) Comparison of two matrix-assisted laser desorption ionization-time of flight mass spectrometry methods with conventional phenotypic identification for routine identification of bacteria to the species level. J Clin Microbiol 48: 1169–1175. doi: 10.1128/JCM.01881-09 20164271 10.1128/JCM.01881-09 PMC2849558

